# Respiratory outcomes of adrenergic beta-antagonists in patients undergoing tracheal extubation: a systematic review and meta-analysis of randomized controlled trials

**DOI:** 10.1016/j.bjane.2025.844659

**Published:** 2025-07-05

**Authors:** Lucas Cael Azevedo Ramos Bendaham, Altair Pereira de Melo Neto, Hilária Saugo Faria, André Richard da Silva Oliveira Filho, Carlos Henrique de Oliveira Ferreira, Marcela da Silva Kazitani Cunha, Victor Gonçalves Soares, Ocílio Ribeiro Gonçalves, Milene Vitória Sampaio Sobral, Mohamed Doma, Denis Maltz Grutcki, Fabrício Tavares Mendonça

**Affiliations:** a Universidade Federal de Roraima, Boa Vista, RR, Brazil; b Universidade Federal de Santa Maria, Santa Maria, RS, Brazil; c Universidade Federal da Paraíba, João Pessoa, PB, Brazil; d Santa Casa de Misericórdia de Marília, Marília, SP, Brazil; e Universidade Federal dos Vales do Jequitinhonha e Mucuri, Diamantina, MG, Brazil; f Universidade Federal do Piauí, Teresina, PI, Brazil; g Universidade do Oeste Paulista, Presidente Prudente, SP, Brazil; h Alexandria Faculty of Medicine, Egypt; i HCA Florida Health Care, Florida, United States of America; j Hospital de Base do Distrito Federal, Departamento de Anestesiologia, Brasilia, DF, Brazil

**Keywords:** Adrenergic beta-antagonists, Airway extubation, Complications, Meta-analysis, Randomized controlled trials

## Abstract

**Background:**

Tracheal extubation after general anesthesia may cause hemodynamic and respiratory complications, with no established strategies to prevent them. We conducted a meta-analysis to evaluate the safety and efficacy of beta-blockers in patients undergoing tracheal extubation.

**Methods:**

We searched the MEDLINE, EMBASE and CENTRAL databases for randomized controlled trials up to 2024 comparing beta-blockers to placebo in patients undergoing tracheal extubation. Primary outcome: cough intensity; secondary: bronchospasm, bucking, hypertension. Risk Ratios (RR) with 95% Confidence Intervals (95% CI) were computed. Leave-one-out sensitivity and meta-regression analyses were performed for outcomes with high heterogeneity.

**Results:**

We included 31 randomized studies, comprising 1,803 patients, of whom 965 received beta-blockers. The risk of moderate/severe cough (RR = 0.21; 95% CI 0.13 to 0.34; p < 0.001; I^2^ = 0%) and hypertension (RR = 0.28; 95% CI 0.13 to 0.58; p < 0.001; I^2^ = 45%) was significantly lower in the beta-blockers group compared with the placebo group. There were no statistically significant differences between groups in the risk of bronchospasm (RR = 0.58; 95% CI 0.17 to 1.94; p = 0.375; I^2^ = 0%) or bucking (RR = 0.47; 95% CI 0.20 to 1.13; p = 0.093; I^2^ = 72%). Sensitivity analysis identified Mendonça (2023) as the main heterogeneity source in bucking.

**Conclusion:**

Our study demonstrates that beta-blockers reduced moderate/severe cough and hypertension in patients undergoing tracheal extubation compared with placebo with no significant difference in the risk of bronchospasm and bucking. These findings suggest beta-blockers may be a valuable strategy for preventing peri-extubation hemodynamic instability and airway hyperresponsiveness.

**Prospero register:**

CRD42024542103.

## Introduction

Extubation is a procedure frequently used in surgeries performed under general anesthesia. Complications from this procedure may affect more than one-third of patients. Cough is a common complication, mainly due to the activation of irritant receptors in the tracheal mucosa, causing a contraction of the smooth muscle in the airways and consequently triggering the cough reflex and bronchospasm.^1^^,^^2^ As a result, there may be an exacerbated hemodynamic responses, leading to cardiovascular and respiratory decompensations.^3^, ^4^, ^5^ This occurs due to the stimulation of the sympathoadrenal reflex, with a concomitant increase in plasma catecholamine levels and activation of alpha and beta-adrenergic receptors.^6^ The development of this response necessitates immediate interventions to reduce the risk of potentially fatal complications such as acute myocardial infarction, arrhythmias, congestive heart failure, and other target organ damage.^6^^,^^7^

Despite these concerns, pharmacological guidelines to control cardiovascular and respiratory decompensations during the peri-extubation period have not yet been developed. In this context, recent studies are investigating the potential use of prophylactic beta-blockers to reduce cardiovascular and respiratory responses and the risk of complications after the procedure.^8^ By counteracting sympathetic activation during acute stress through their antagonistic action on beta-1 receptors, these medications may prevent a hyperdynamic state throughout the tracheal extubation phase without prolonging the recovery phases.^9^, ^10^, ^11^, ^12^

The efficacy and safety of beta-blockers during tracheal extubation remain uncertain.^12^, ^13^, ^14^, ^15^, ^16^, ^17^, ^18^, ^19^, ^20^, ^21^, ^22^, ^23^, ^24^, ^25^, ^26^, ^27^, ^28^, ^29^, ^30^, ^31^, ^32^, ^33^, ^34^, ^35^, ^36^, ^37^, ^38^, ^39^, ^40^, ^41^, ^42^ Individual trials lack sufficient power to detect significant differences in outcomes and adverse events. To address these limitations, this meta-analysis pools data from multiple Randomized Controlled Trials (RCTs) to enhance statistical power and provide robust conclusions on the efficacy and safety of beta-blockers in tracheal extubation.

## Material and methods

This systematic review and meta-analysis were conducted in accordance with the Cochrane Collaboration and the Preferred Reporting Items for Systematic Reviews and Meta-Analysis (PRISMA) statement guidelines and followed the methodological recommendations outlined in the Cochrane Handbook for Systematic Reviews of Interventions.^43^^,^^44^ The protocol of this study was prospectively registered in the International Prospective Register of Systematic Reviews (PROSPERO; CRD42024542103).

### Search strategy and data extraction

We systematically searched in databases of MEDLINE, EMBASE and the Cochrane Central Register of Controlled Trials (CENTRAL) from inception to April 25^th^, 2024, with the search terms presented in Supplementary Table 1. The titles and abstracts were first reviewed. Studies that did not satisfy the inclusion criteria were excluded, and all abstracts deemed potentially eligible were obtained in full text and assessed to confirm their inclusion. The entire screening process was conducted independently by two authors (L.B. and A.N.), followed by a comparison of decisions. Any discrepancies in decisions were resolved by a third independent author. Four authors (A.F., M.C., H.F. and C.F.) independently organized and extracted the data, using standardized tables for accuracy, following predefined search criteria and quality assessment. Disagreements were resolved by consensus between the authors.

### Eligibility criteria

We included studies that fulfilled the following eligibility criteria: (1) Randomized Controlled Trials (RCTs) published in the indexed databases; (2) Studies comparing adrenergic beta-antagonists with placebo; (3) trials involving patients undergoing tracheal extubation; and (4) Studies assessing extubation-related complications using validated clinical scales or predefined hemodynamic parameters. We excluded studies based on the following criteria: (1) Lack of a control group; (2) Overlapping patient populations; (3) Trials not involving patients undergoing tracheal extubation; and (4) Administration of the drug solely during anesthetic induction, with no relevance to tracheal extubation outcomes.

### Outcomes and subgroups

Primary outcomes were the incidence of cough and its severity, classified as no/mild cough, and moderate/severe cough. Secondary outcomes included Systolic Blood Pressure (SBP), Diastolic Blood Pressure (DBP), Mean Arterial Pressure (MAP), Mean Heart Rate (MHR), risk of bronchospasm, hypertension, hypotension, tachycardia, bradycardia, postoperative nausea or vomiting, and bucking, defined as a situation in which a patient is trying to cough and strain on an endotracheal tube and has violent expiratory contraction of skeletal muscles secondary to endotracheal tube stimulation of the tracheal mucosa.

Sub-analyses included data restricted to the time of outcome measurement after extubation (at extubation, 1-minute, 2 minutes, 5 minutes, 10 minutes, and 15 minutes or more).

### Quality assessment

Quality assessment of RCTs was performed using the Cochrane Collaboration’s tool for assessing the risk of Bias in Randomized trials (RoB-2). Studies were scored as high, low, or unclear risk of bias in 5 domains: selection, performance, detection, attrition, and reporting biases.^45^ Bias risk assessment was conducted independently by two authors (A.N and O.G.). Discrepancies were resolved through consensus among the authors. Publication bias was assessed with contour-enhanced funnel plot analysis^46^ and Egger’s test^47^ of efficacy endpoints and evaluation for symmetrical distribution of trials with similar weights, using the Pustejovsky and Rodgers^48^ approach when the standardized mean difference was used for the outcome of interest.

### Statistical analysis

Treatment effects were compared for binary outcomes using Risk Ratios (RR) with 95% Confidence Intervals (95% CI). Mean Differences (MD) with 95% CI were used to compare the treatment effects for continuous endpoints. Given the expected heterogeneity between studies, we adopted the DerSimonian and Laird random-effects model for all outcomes reported. We used the Cochrane Q test and I^2^ statistics to assess heterogeneity; p-values inferior to 0.1 and I^2^ > 40% were considered significant for heterogeneity.^43^ The p-values inferior to 0.05 were considered statistically significant. Funnel Plots with Egger’s test was used to address publication bias in every outcome and subgroup that had at least 10 studies. R version 4.4.0 and the “meta” extension package was used for all analyses.^49^

### Sensitivity analysis

We performed a pre-specified sensitivity analysis for primary endpoints with (1) A leave-one-out approach to ensure that results were not dependent on a single study and to evaluate studies that had high contributions to the heterogeneity on primary endpoints when I^2^ ≥ 40; (2) Several univariable meta-regression analyses to assess any interactions with some covariates (time of drug administration; type of adrenergic beta-antagonist; age; American Society of Anesthesiologists physical status classification; type of surgery; preanesthetic medication; type of general anesthesia; baseline SBP, DBP, MAP, and MHR; duration of surgery and anesthesia) for the continuous outcomes reported by at least 9 studies. A multivariable meta-regression was not conducted to assess the robustness and validity of the findings due to the limited number of included studies and lack of statistical significance of multiple covariates. Current methodological guidelines recommend a minimum of 10 studies per covariate to ensure reliable estimates in multivariable meta-regression analyses.^43^

## Results

### Study selection and characteristics

**Figure 1:**
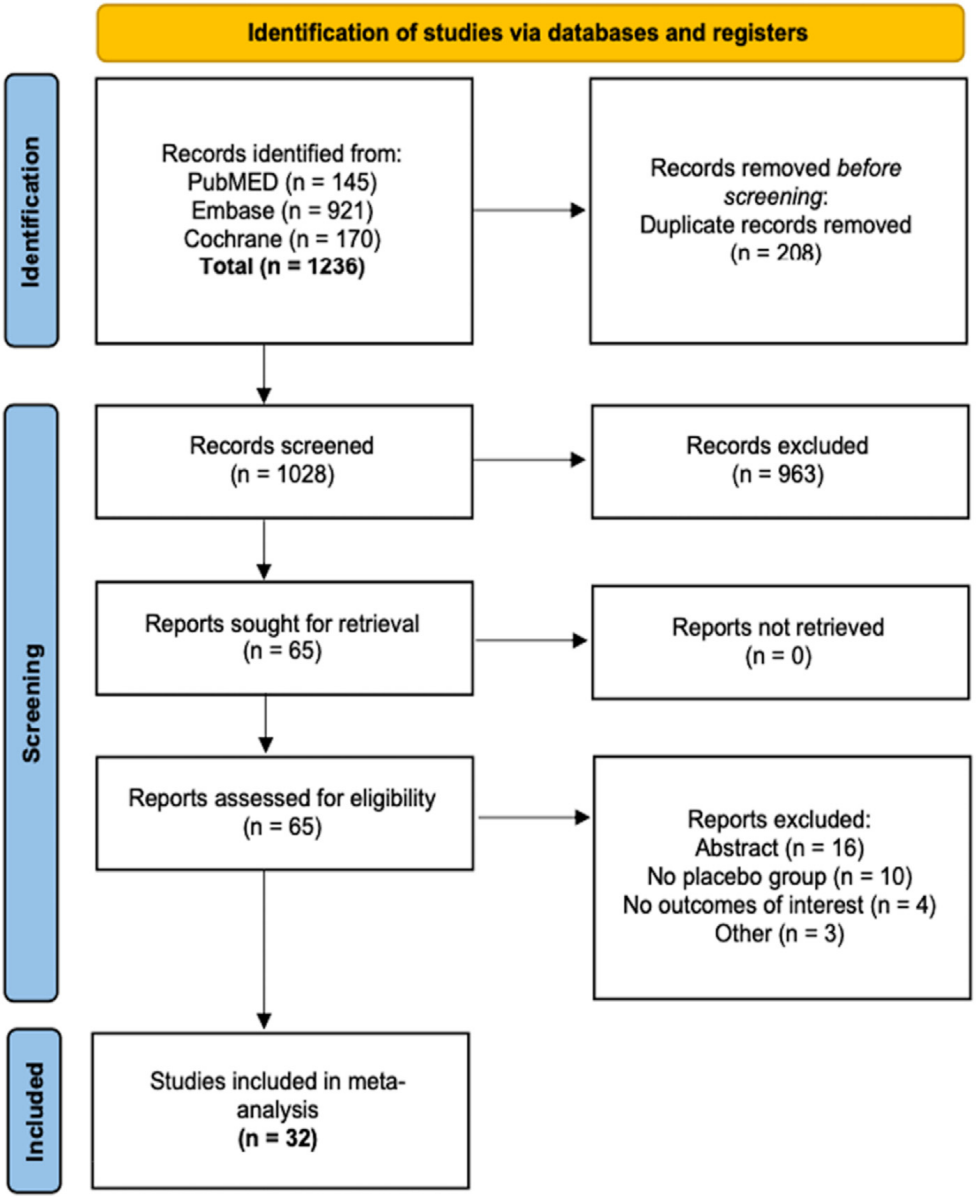
PRISMA flow diagram of study screening and selection.

As outlined in Figure 1, this study included 31 RCTs with a total of 1,803 patients, of whom 965 (53.5%) were assigned to the beta-blockers group and 838 (46.5%) to the placebo group.^12^, ^13^, ^14^, ^15^, ^16^, ^17^, ^18^, ^19^, ^20^, ^21^, ^22^, ^23^, ^24^, ^25^, ^26^, ^27^, ^28^, ^29^, ^30^, ^31^, ^32^, ^33^, ^34^, ^35^, ^36^, ^37^, ^38^, ^39^, ^40^, ^41^, ^42^ Among the interventions, 21 studies used esmolol, 4 labetalol, 3 used metoprolol, 1 landiolol, 1 atenolol, and 1 propranolol. The mean age of patients across studies ranged from 31.2 to 69 years, and the percentage of female patients ranged from 14.28% to 100%. At baseline, MAP ranged from 30.05 to 124.20 and MHR ranged from 52.74 to 98.76. Detailed baseline characteristics of the included studies can be found in Table 1. Given the heterogeneity in pharmacologic properties and clinical application of these agents, we present in Supplementary Table 2 a descriptive summary of their key characteristics and relevance in the context of tracheal extubation based on the studies included in this meta-analysis.

**Table 1:** Baseline characteristics of included studies.

Study	Country	Intervention	Patients, n (I/C)	Female, % (I/C)	Age^a^, Y (I/C)	Follow-up	ASA classification	Preanesthetic medication	Weight. Kg^a^ (I/C)
Queiroz 2024	Brazil	Metoprolol (5 mg/20 mL)	102/105	52 / 58	43.6 (15.1) / 47.1 (17)	120 min after extubation	I. II or III	Intravenous midazolam (0.05 mg.kg^-1^)	72.6 (14.6) / 69.8 (12.8)
Mendonça 2023	Brazil	Esmolol (2 mg.kg^-1^)	45/45	53.3 / 64.4	49.2 (12.8) / 44.2 (15.1)	10 min after extubation	I. II or III	Intravenous midazolam (0.05 mg.kg^-1^)	68.9 (10.3) / 68.7 (9.7)
Alkaya 2014	USA	Esmolol (0.2 μg.kg^-1^.min^-1^)	15/15	43.7 / 43.8	39.4 (10.7) / 45.0 (13.3)	10 min after extubation	I or II	Intravenous midazolam (0.05 mg.kg^-1^)	NA
Arar 2007	USA	Labetalol (0.15‒0.3 mg.kg^-1^)	40/40	57.5 / 60	57.4 (8.0) / 59.2 (9.9)	NA	I	NA	70.0 (15.3) / 70.6 (10.2)
Hosseinzadeh 2013	Iran	Esmolol (infusion at 0.5 mg.kg^-1^ 4 min before the extubation. followed by an infusion at 0.15 mg.kg^-1^.min^-1^ for 10 min after extubation)	30/30	50/70	46.0 (16.2) / 49.0 (17)	15 min after extubation	I or II	NA	73.2 (10.3) / 71.6 (12.0)
Chia 2004	Taiwan	Esmolol (0.2 μg.kg^-1^.min^-1^)	49/48	NA	48.5 (30–79) / 49.8 (27–75)^b^	PO + 72 h	I or II	NA	57.4 (7.2) / 61.3 (10.6)
Grillo 2003	France	Esmolol (0.3 mg.kg^-1^.min^-1^)	15/15	NA	52 (10) / 47 (15)	60 min after anesthesia with esmolol	I or II	NA	65 (10) / 68 (16)
O'Dwyer 1993	UK	Esmolol (500 ng.kg^-1^ over l min followed by 100 ng.kg^-1^.min^-1^)	7/7	NA	59.7 (5) / 58.8 (3.5)	10 min after extubation	NA	Etomidate (fentanyl 5 μg. kg^-1^) and midazolam	74.4 (3) / 82.8 (5.6)
Kawaguchi. 2010	Japan	Landiolol (0.125 mg.kg^-1^.min^-1^ followed by an infusion at 0.01–0.04 mg.kg^-1^.min^-1^)	15/15	26.7/20	60 (10) / 59 (8)	10 min after extubation	NA	NA	58 (9) / 60 (9)
Elokda 2015	Arabi Saudita	Esmolol (1 mg.kg^-1^ over 30 s followed 100 μg.kg^-1^.min^-1^)	50/50	55/60	60 (3) / 62 (4)	10 min after extubation	I or II	Bromazepam (3 mg) and ranitidine (150 mg)	82.6 (6) / 85 (10)
Kshama 2022	India	Esmolol (0.5 mg.kg^-1^ and 1 mg.kg^-1^)	40/20	15/40	39.3 (8.4) / 38.7 (11.9)	10 min after extubation	NA	NA	62.8 (9.3) / 61.3 (7.9)
Song 2021	China	Esmolol (0.5 mg.kg^-1^ and 1.0 mg.kg^-1^)	84/41	38/39	56 (10.9) / 56.9 (8.9)	5 min after extubation	I. II or III	NA	67.4 (9.7) / 70.9 (10.6)
Shetabi 2023	Iran	Labetalol (0.1 mg.kg^-1^ or 0.2 mg.kg^-1^)	48/24	58.3/79.2	31.8 (10.7) / 33 (14.7)	10 min after extubation	I or II	NA	72.0 (8.3) / 69.9 (9.8)
Dash 2023	India	Esmolol (1 mg.kg^-1^)	30/30	53.3/46.6	37.1 (12.0) / 38.9 (12.5)	30 min after extubation	I or II	NA	61.5 (11.4) / 65.4 (10.9)
Lim 2000	Singapore	Esmolol (500 μg. kg^-1^. followed by esmolol infusions at 100 μg.kg^-1^.min^-1^ or 200 μg.kg^-1^.min^-1^)	24/12	45.8/66.7	NA	5 min after extubation	I or II	NA	NA
Maharjan 2005	Nepal	Propranolol (1 mg or 0.5 mg)	42/21	19.0/14.3	44.9 (15.8) / 35.8 (11.0)	PO + 6 h	I or II	Diazepam (5 mg), ranitidine (150 mg), and metoclopramide (10 mg)	56.9 (8.9) / 60.9 (9.9)
Morais 2020	Brazil	Esmolol (0.5 mg.kg^-1^ bolus followed by an infusion at 15 μg.kg^-1^.min^-1^)	20/20	85/85	35.8 (10.9) / 33.2 (8.7)	PO + 24 h	II or III	Dipyrone (2 mg) and parecoxib (40 mg)	105.6 (20.2) / 109.8 (11.2)
Radwan 2016	Egypt	Labetalol (infusion in a rate of 0.5 mg.kg^-1^.hr^-1^)	25/25	44/37	44 (6) / 40 (11)	PO + 4 h	I or II	Ranitidine (50 mg), metoclopropramide (10 mg), and dexamethasone (0.15 mg.kg^-1^)	80 (10) / 79 (11)
Sohn 1995	South Korea	Esmolol (1 mg.kg^-1^)	30/30	43.3/40	36.3 (15.1) / 39.4 (16.4)	4 min after extubation	I or II	Midazolam (0.05 mg.kg^-1^)	58.6 (6.9) / 58.5 (8.3)
Felding 1994	Denmark	Metoprolol (0.07 mg.kg^-1^)	10/10	NA	56 (10) / 54 (12)	PO + 180 min after extubation	NA	Diazepam (0.2 mg.kg^-1^)	NA
Velayutham 2020	India	Atenolol (50 mg)	25/25	24/32	39 (11) / 38 (7)	PO + 12 h	I. II or III	Diazepam (10 mg)	61 (7) / 63 (5)
Vandenberg 1997	Saudi Arabia	Esmolol (4 mg.kg^-1^)	20/20	45/50	68/64	NA	I. II or III	Temazepam (10 mg) VO	64/63
Unal 2008	Turkey	Esmolol (0.1 mg.kg^-1^.min^-1^ or 0.2 mg.kg^-1^.min^-1^)	30/15	46.7/53.3	49.2 (16.4) / 50.3 (12.2)	30 min after extubation	I or II	NA	165.8(9.3) / 77.5(9.3)
Ersin 2005	Turkey	Esmolol (bolus dose at 1.5 mg.kg^-1^ for 30 seconds)	15/15	60/60	41.7 (12.8) / 38.5 (12.2)	10 min after extubation	I or II	NA	72.5(11) / 68.7(13.3)
Yorugloku 1999	Turkey	Metoprolol (0.02 mg.kg^-1^)	15/15	NA	46 (6) / 41 (6)	5 min after extubation	I or II	Intramuscular atropine and pethidine	77(9) / 70(8)
Amar 1991	USA	Labetalol (infusion at 0.15 mg.kg^-1^ intravenous. followed by 0.25‒0.3 mg.kg^-1^ every 3 min as needed)	8/8	100/100	43.9 (6.7) / 35.9 (10.3)	NA	I	Intravenous midazolam (0.5 mg)	70.0 (15.3) / 70.6 (10.2)
Zhang 2017	China	Esmolol (continuous perfusion at a dose of 50 μg.kg^-1^.min^-1^ during operation and infusion at a dose of 0.3 mg.kg^-1^ 3 min before tracheal intubation)	30/30	40/43.3	69.3 (5.4) / 66.1 (12.5)	30 min after extubation	I or II	NA	60.7(6.7) / 60.3(7.2)
Kurian 2008	United Kingdom	Esmolol (infusion of esmolol at 0-300 μg.kg^-1^.min^-1^)	31/37	19.3/10.8	60.2 (6.7) / 61.1 (7.5)	180 min after extubation	NA	Lorazepam (2‒3 mg)	82.5 (13.8) / 86.0 (14.1)
Nam 1996	South Korea	Esmolol (infusion at 1.5 mg.kg^-1^ 2 min before tracheal extubation)	20/20	NA	31.2 (9.6) / 33.2 (7.5)	5 min after extubation	I	Glycopyrrolate (0.2 mg) and intramuscular triflupromazine HCL (15 mg)	69.1(9.4) / 64.8(8.5)
Zeng 2007	China	Esmolol (bolus at 0.5 mg.kg^-1^ for 5 min. followed by an infusion at 50 μg.kg^-1^.min^-1^ until the end of surgery)	20/20	75/65	NA	5 min after extubation	I or II	NA	58(13)/57(10)
Lee 2010	South Korea	Esmolol (bolus at 1.0 mg.kg^-1^ followed by an infusion of 10 μg.kg^-1^.min^-1^)	30/30	60/53.3	61.7 (6.3) / 58.6 (6.6)	PO + 24 h	I or II	Glycopyrrolate (0.2 mg)	161.8 (6.6) / 160.4 (6.3)

Study	Type of general anesthesia	Height^c^, cm (I/C)	SBP^c^, mmHg (I/C)	DBP^c^, mmHg (I/C)	MAP^c^, mmHg (I/C)	MHR^c^, beats/min (I/C)	Duration of surgery^c^, min (I/C)	Duration of anesthesia^c^, min (I/C)	
Queiroz 2024	Balanced anesthesia or total intravenous anesthesia	NA	NA	NA	94.2/94.3	82.4/79.2	NA	NA	
Mendonça 2023	Balanced anesthesia	165 (10) / 167 (10)	110.4 (15.1) / 108.0 (13.4)	NA	NA	77.8 (10.1) / 74.3 (10.2)	NA	NA	
Alkaya 2014	Balanced anesthesia	NA	137/137	85/85	105/105	80/85	213.5 (78.2) / 220.9 (101.8)	203.7 (78.5) / 211.1 (102.4)	
Arar 2007	Balanced anesthesia	NA	142.6 (15.5) / 140.7 (19.5)	76.4 (11.3) / 74.0 (10.7)	98.0 (10.2) / 96.6 (10.9)	98.8 (10.5) / 96.7 (13.0)	288 (43.6) / 298.2 (45.6)	314.1 (47.2) / 329.6 (50.9)	
Hosseinzadeh 2013	Balanced anesthesia	NA	17.6 (109.7) / 14.9 (115.6)	13.5 (69.7) / 13.1 (72.0)	NA	NA	184.6 (42.7) / 186.6 (62.6)	NA	
Chia 2004	Balanced anesthesia	153.6 (5.4) / 155.6 (4.4)	NA	NA	87.6 (6.1) / 84.8 (6.1)	74.2 (7.8) / 71.6 (9.2)	122 (54) / 138 (50)	NA	
Grillo 2003	Balanced anesthesia	165 (6) / 168 (6)	NA	NA	95 (12) / 88 (15)	76 (9) / 77 (7)	NA	NA	
O'Dwyer 1993	Balanced anesthesia	NA	141 (10) / 135 (6.2)	77.8 (4.1) / 79.8 (3)	74.3 (5.2) / 89 (7.1)	70 (2) / 69 (3.7)	NA	NA	
Elokda. 2015	Balanced anesthesia	165 (10) /166 (8)	130/131	73.0/71.5	62.1/30.0	81.2 / 81.2	60 (5) / 61.6 (8)	65.8 (7) / 63 (8)	
Kshama 2022	Balanced anesthesia	158.55 (7.3) / 157.7 (6.6)	136.7 (14.7) / 130 (16.2)	86.0 (10.1) / 82.5 (12.9)	103.7 (12.2) / 98.8 (13.2)	96.3 (14) / 90.4 (10.1)	NA	NA	
Vandenberg 1997	Balanced anesthesia	NA	NA	NA	NA	NA	34/24	NA	
Song 2021	Balanced anesthesia	168.4 (7.4) / 170.0 (8.9)	NA	NA	104.9 (12.1) / 105.8 (11.3)	52.7 (21.4) / 57.3 (24.2)	216.3 (35.0) / 212.8 (26.1)	NA	
Shetabi 2023	Balanced anesthesia	NA	121.2 (9.6) / 122 (8.8)	74.1 (6.2) / 77 (9.6)	104.9 (4.0) / 106.2 (8.6)	80.8 (9.2) / 85.6 (6.4)	NA	NA	
Dash 2023	Balanced anesthesia	164.8 (9.9) / 163.8 (9.4)	119.9 (8.0) / 122.5 (11.0)	77.7 (6.0) / 75.7 (8.5)	91.7 (4.7) / 91.3 (6.4)	71.8 (4.5) / 75.2 (7.7)	NA	NA	
Lim 2000	Balanced anesthesia	NA	131.5 (18.4) / 129 (15)	NA	NA	71.4 (7.4) / 70.8 (11.9)	NA	NA	
Maharjan 2005	Balanced anesthesia	NA	NA	NA	102.5 (10.5) / 106.6 (12.7)	89.4 (23.4) / 95.6 (12.3)	72.7 (32.8) / 80.5 (19.2)	81.4 (30.3) / 91.4 (22.2)	
Morais 2020	Balanced anesthesia	161.9 (8.0) / 164.4 (9.7)	NA	NA	NA	NA	104.3 (14.3) / 112.8 (12.5)	NA	
Radwan 2016	Balanced anesthesia	170 (8) / 164 (6)	NA	NA	NA	NA	247 (74) / 243 (69)	NA	
Sohn 1995	Balanced anesthesia	58.5 (8.3) / 58.6 (6.9)	125.2(15.76) / 130.25(19.2)	NA	NA	77.5 (9.1) / 82.7 (13.9)	175.5 (84.9) / 182.8 (73.1)	NA	
Felding 1994	Balanced anesthesia	NA	NA	NA	91 (17) / 93 (15)	NA	NA	NA	
Velayutham 2020	Balanced anesthesia	NA	NA	NA	92 (6) / 94 (5)	83 (9) / 81 (8)	NA	NA	
Unal 2008	Balanced anesthesia	165.8 (9.3) / 168.3 (8.2)	NA	NA	124.2 (20.1) / 117.4 (9.4)	82.5 (9.9) / 89.4 (10.0)	134.1 (74.5) / 121.2 (62.8)	156.9 (74.4) / 145.5 (69.4)	
Ersin 2005	Balanced anesthesia	NA	132.1 (9.2) / 133.1 (10.4)	78.3 (7.6) / 79 (6.1)	NA	87.2 (7.2) / 86.8 (8.2)	119.4 (5.7) / 117.4 (7.8)	137.7 (6.1) / 136.2 (8.1)	
Yorugloku 1999	Balanced anesthesia	NA	126.96 (12.38) / 122.83 (12.38)	75.3 (10) / 85 (12.1)	89.7 (8.1) / 91.1 (8.4)	92.2 (7.4) / 88.7 (10.5)	88 (12) / 92 (6)	NA	
Lee 2010	Total intravenous anesthesia	161.8 (6.6) / 160.4 (6.3)	NA	NA	89.8 (13.3) / 90.2 (12.2)	71.6 (9.5) / 72.3 (11.2)	42.5 (4.8) / 41.3 (7.2)	57.5 (2.8) / 56.3 (5.2)	
Amar 1991	Balanced anesthesia	NA	130.23/124.20	70.5/68.9	93.9 / 91.6	82.4 / 81.3	120.0 (29.7) / 125.6 (50.0)	160.6 (34.0) / 159.4 (51.8)	
Zhang 2017	Total intravenous anesthesia	NA	NA	NA	92.7 (7.7) / 95.9 (6.4)	76.8 (6.5) / 73.7 (8.2)	180.5 (16.5) / 180.3 (18.2)	NA	
Kurian 2008	Balanced or Total Intravenous anesthesia	170.7 (8.0) / 173.1 (7.8)	119.56 (4.15) / 122.57 (3.53)	NA	NA	86.1 (2.2) / 95.3 (2.2)	NA	NA	
Nam 1996	Balanced anesthesia	NA	121 (7.1) / 118 (6.2)	77 (5.3) / 78 (4.3)	NA	79 (7.3) / 80 (5.9)	45 (15.6) / 38 (20.4)	NA	
Zeng 2007	Total intravenous anesthesia	159 (8) / 160 (9)	112.9 (9) / 117.8 (9.6)	77.5 (7.1) / 78.4 (8)	NA	85 (6.6) / 88.8 (10.3)	88 (3) / 74 (28)	102 (31) / 92 (3)	
Kawaguchi 2010	Balanced anesthesia	159 (7)/ 162 (7)	148 (19) / 150 (19)	85 (11) / 85 (10)	63.6 (6.3) / 64.1 (9.1)	72 (11) / 70 (14)	228 (82) / 251 (108)	309 (83) / 331 (126)	

a Mean (standard deviation).

b Mean (range); n, Number; y, Years; kg, Kilogram; mg, Miligram; n, Nanogram; μ, Microgram; mL, Militer; min: minutes; h, Hours; s, Seconds; ASA, American Society of Anesthesiologists classification; I/C, Intervention group/Control group; PO, Post-Operatory; NA, Not Available.

c Mean (standard deviation); cm, Centimeter; mmHg, Millimeter of mercury; min, Minutes; I/C, Intervention group/Control group; NA, Not Available; SBP, Systolic Blood Pressure; DBP, Diastolic Blood Pressure; MAP, Mean Arterial Pressure; HR, Heart Rate.

### Pooled analysis

#### Primary outcomes

**Figure 2:**
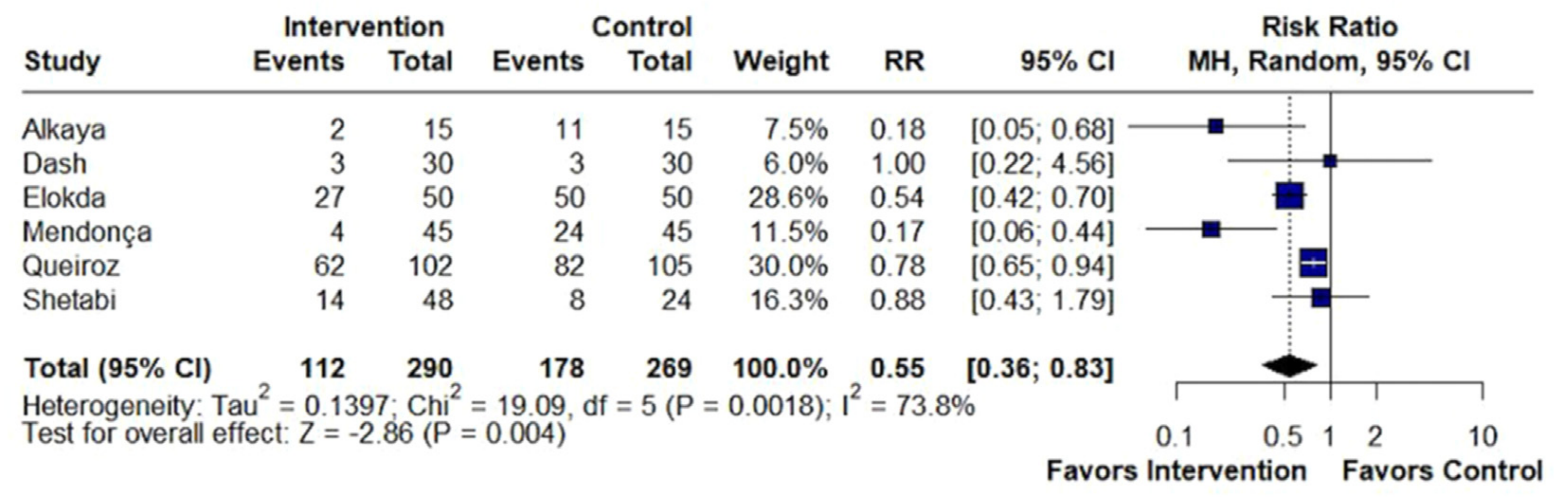
Beta-blockers significantly reduced the incidence of cough in patients undergoing tracheal extubation compared with placebo. MH, Mantel-Haenszel; CI, Confidence Interval.

**Figure 3:**
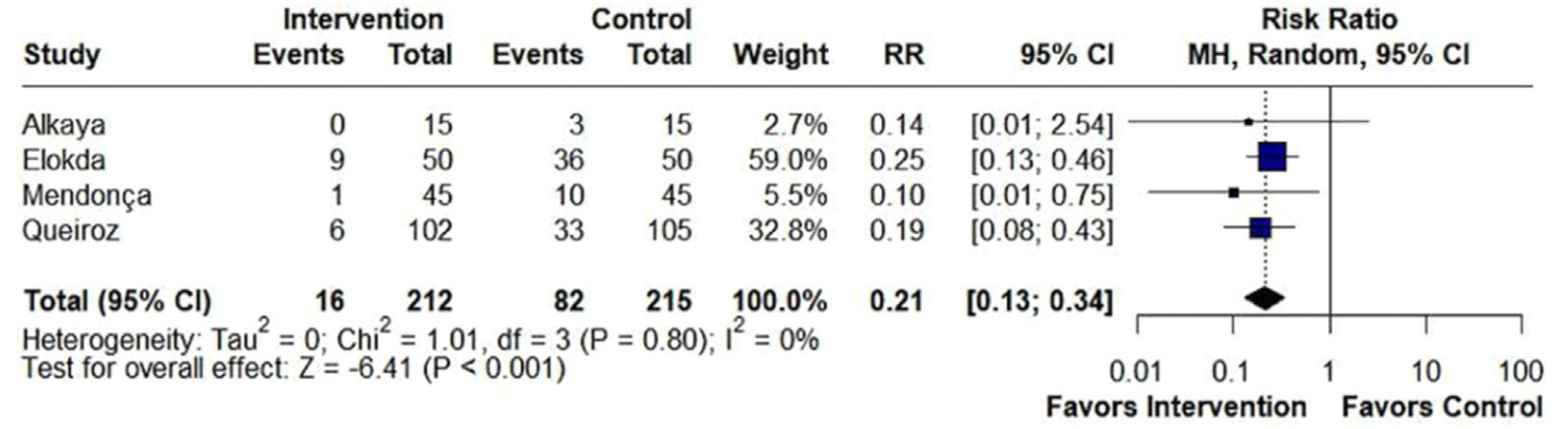
Beta-blockers significantly reduced the incidence of moderate/severe cough in patients undergoing tracheal extubation compared with placebo. MH, Mantel-Haenszel; CI, Confidence Interval.

**Figure 4:**
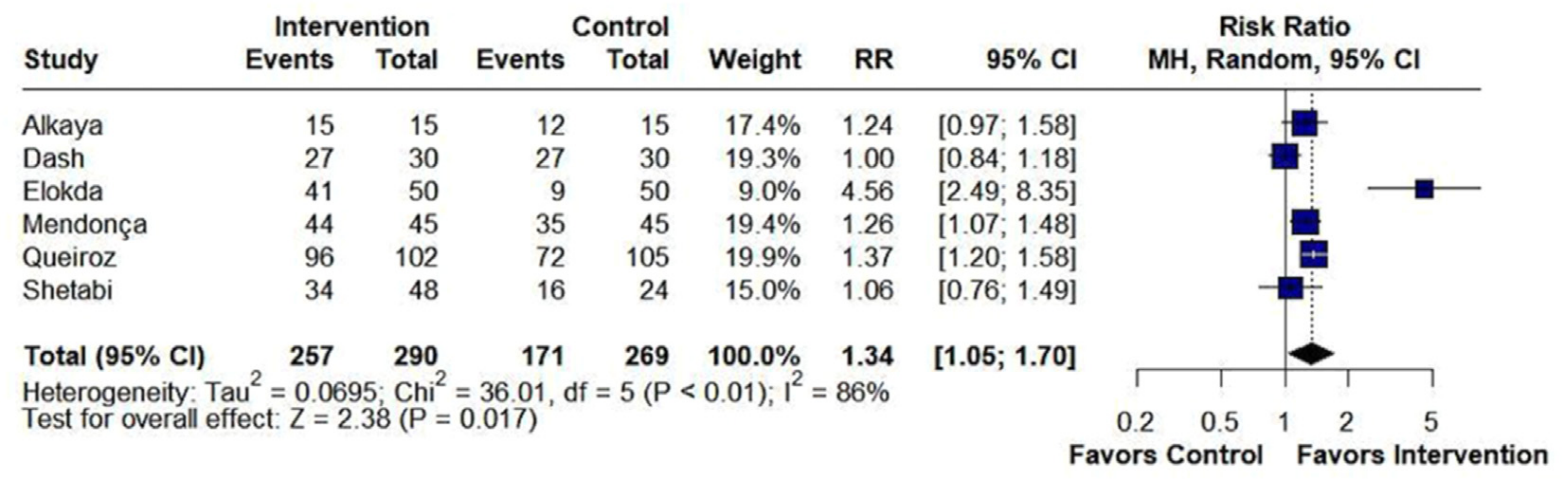
Beta-blockers significantly increased the incidence of no/mild cough in patients undergoing tracheal extubation. MH, Mantel-Haenszel; CI, Confidence Interval.

Beta-blockers significantly reduced the incidence of cough (RR = 0.55; 95% CI 0.36 to 0.83; p < 0.01; I^2^ = 73%; Fig. 2). Also, beta-blockers significantly altered the distribution of cough severity during tracheal extubation, shifting the severity distribution from moderate/severe to none/mild. Specifically, they significantly reduced the incidence of moderate/severe cough (RR = 0.21; 95% CI 0.13 to 0.34; p < 0.01; I^2^ = 0%; Fig. 3), while simultaneously increasing the incidence of patients experiencing no/mild cough (RR = 1.34; 95% CI 1.05 to 1.70; p = 0.017; I^2^ = 86%; Fig. 4).

#### Secondary outcomes

**Figure 5:**
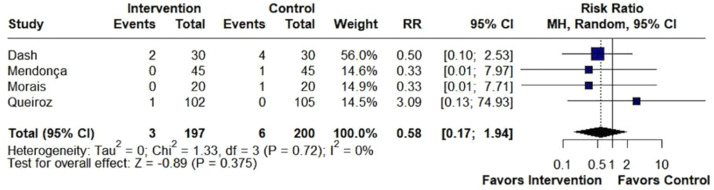
There was no difference between groups in the incidence of bronchospasm in patients undergoing tracheal extubation. MH, Mantel-Haenszel; CI, Confidence Interval.

**Figure 6:**
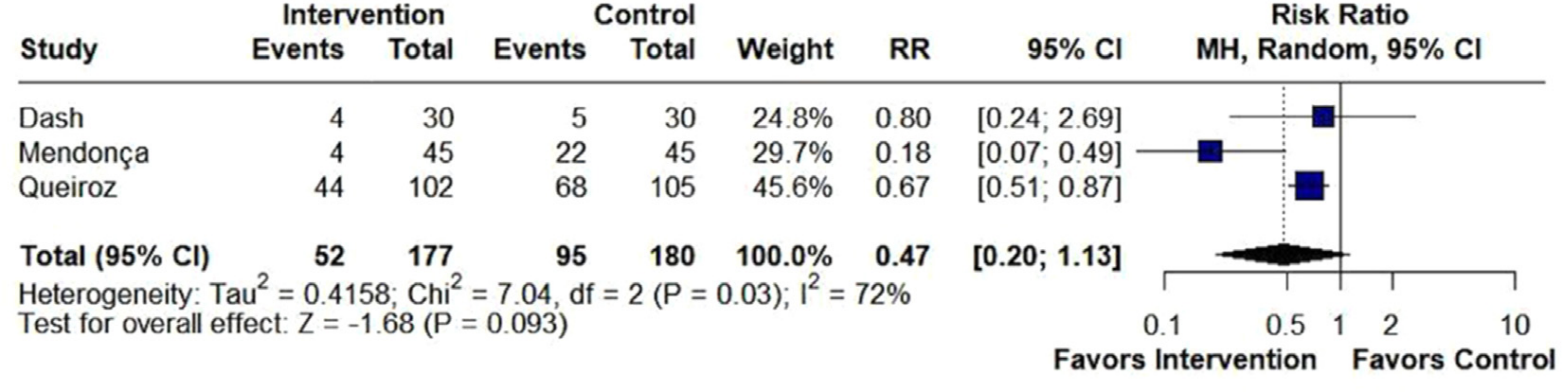
There was no difference between groups in the incidence of bucking in patients undergoing tracheal extubation. MH, Mantel-Haenszel; CI, Confidence Interval.

There was no difference between groups in bronchospasm (RR = 0.58; 95% CI 0.17 to 1.94; p = 0.375; I^2^ = 0%; Fig. 5), bucking (RR = 0.47; 95% CI 0.20 to 1.13; p = 0.093; I^2^ = 72%; Fig. 6), hypotension (RR = 1.43; 95% CI 0.87 to 2.38; p = 0.161; I^2^ = 0%; Supplementary Fig. 1) and bradycardia (RR = 1.24; 95% CI 0.31 to 4.97; p = 0.759; I^2^ = 0%; Supplementary Fig. 2).

The risk of hypertension (RR = 0.28; 95% CI 0.13 to 0.58; p < 0.001; I^2^ = 45%; Supplementary Fig. 3), tachycardia (RR = 0.20; 95% CI 0.08 to 0.51; p < 0.001; I^2^ = 71%; Supplementary Fig. 4), and nausea or vomiting (RR = 0.60; 95% CI 0.50 to 0.72; p < 0.001; I^2^ = 2%; Supplementary Fig. 5) was significantly reduced in the beta-blocker group compared with the placebo group in patients undergoing tracheal extubation.

#### Hemodynamic variables

SBP, DBP, MAP, and MHR were significantly lower in the beta-blockers group compared with the placebo group at tracheal extubation after 1, 2, 5, 10, and 15 or more minutes (Supplementary Table 2). However, there was no significant difference between groups for MAP and MHR at tracheal extubation after 15 minutes or more.

### Sensitivity analyses

We conducted leave-one-out sensitivity analyses for the outcomes of bucking, hypertension, cough, no/mild cough, moderate/severe cough, and tachycardia due to high heterogeneity. The leave-one-out analysis of the outcome of bucking showed that omitting Mendonça (2023)^12^ led to a significantly lower incidence of bucking in the beta-blockers group compared with the placebo group, with no heterogeneity observed (I^2^ = 0%; Supplementary Fig. 6). For the outcome of hypertension, cough, and no/mild cough, no study was identified as driving the heterogeneity, all omissions remained with high heterogeneity, favoring beta-blockers (Supplementary Fig. 7; Supplementary Fig. 8; Supplementary Fig. 9). The leave-one-out sensitivity analysis for the outcome of tachycardia showed similar results in all scenarios with a lower heterogeneity when Shetabi (2023)^35^ is omitted (I^2^ = 18%; Supplementary Fig. 10).

Additionally, we performed a meta-regression analysis for the following outcomes: SBP, DBP, MAP, and MHR using the mean age and the mean values at baseline of SBP, DBP, MAP, and MHR as predictors. Results are presented in Supplementary Table 4. Our results showed that age was a significant predictor (QMp < 0.05) for MHR at tracheal extubation, with higher baseline age values resulting in more positive mean differences, favoring placebo over beta-blockers. SBP, DBP, MAP, and MHR were not significant predictors of any outcome. Significant heterogeneity remained after accounting for the moderator effects of the selected predictors.

### Quality assessment

The individual RCT appraisal is reported in Supplementary Table 5. Sixteen studies [12, 13, 14, 15, 16, 17, 18, 19, 20, 21, 22,25,32,34,39,41,50] were considered at moderate risk of bias. Twelve studies presented moderate bias in bias from randomization process, nine in bias due to deviations from intended interventions, three in bias in measurement of the outcomes and twenty-two in the selection of the reported result. Nine RCTs were considered at high risk of bias and the others were classified as low risk of bias.

Publication bias was investigated for the outcomes of SBP, DBP, MAP, and MHR for every subgroup that had at least 10 studies (Supplementary Fig. 11). The visual inspection of the funnel plots with enhanced contour showed no visible signs of the “small study effect” with symmetrical funnel plots for most of the subgroups. This finding is corroborated by the results of the Egger’s Test (Supplementary Table 6).

## Discussion

In this systematic review and meta-analysis of 31 RCTs, including 1,803 patients, we compared the use of beta-blockers with placebo in preventing complications in patients undergoing tracheal extubation. The main findings from the pooled analysis were: (1) The use of beta-blockers was associated with a reduced risk and intensity of cough; (2) The risk of hypertension, tachycardia and nausea or vomiting was significantly reduced in the beta-blocker group compared with the placebo group.

About 70% of patients undergoing procedures requiring general anesthesia and tracheal intubation may experience coughing.^51^ Coughing during tracheal extubation can lead to significant complications for patients, such as hypertension, tachycardia, myocardial ischemia, surgical bleeding, laryngospasm, bronchospasm, and increased intracranial and intraocular pressure.^5^ There is evidence that beta-blocker reduce the incidence of coughing in these patients by blocking ion channels, particularly voltage-dependent sodium channels and L-type calcium channels, in unmyelinated C fibers of vagal afferent nerves that innervate the upper airway and proximal bronchioles, thereby reducing excitability during procedures such as orotracheal intubation and extubation.^52^, ^53^, ^54^, ^55^

This meta-analysis showed that approximately 11% of patients receiving beta-blocker experienced significant coughing (moderate/severe intensity) during the peri-extubation period, compared to an incidence of 36% among those who received placebo. These findings are consistent with individual data from RCTs that investigated the incidence of this outcome in the population in question.^12^^,^^18^^,^^33^ Thus, it is evident that beta-blockers may be a promising alternative to prevent cough and reduce complications during tracheal extubation.

Moreover, the effectiveness of beta-blockers in reducing bucking can be attributed to their ability to block the effects of the sympathetic nervous system, specifically by antagonizing beta-adrenergic receptors.^56^ This inhibition leads to a decrease in heart rate, blood pressure, and overall sympathetic output, which can calm reflexive responses, such as coughing or bucking, particularly during anesthesia or intubation.^57^ By reducing the surge of adrenaline, beta-blockers helps stabilize cardiovascular and respiratory functions, minimizing involuntary movements that could disrupt medical procedures.^58^ Queiroz (2024)^33^ and Mendonça (2023)^12^ showed significantly lower risk in the beta-blocker group, and Dash (2023)^17^ indicated lower risk but with no statistical difference, suggesting that the intervention may be effective in reducing bucking. However, the limited number of patients led to Queiroz's (2024)^33^ results dominating the analysis. Thus, more RCTs evaluating bucking are needed to reach a more robust conclusion.

Furthermore, cardioselective beta-blockers, such as metoprolol, block the β1-adrenoceptor, leaving the β2-adrenoceptor free in the adrenergic response during extubation, which may help to prevent bronchospasm in this group of patients.^59^ Our data indicated a slight trend toward a reduction in the incidence of bronchospasm in the beta-blocker group, with a 48% lower relative risk of this complication in patients who received beta-blocker compared to those who received placebo. However, neither the individual studies^12^^,^^17^^,^^30^^,^^33^ nor the pooled analysis results were statistically significant. Therefore, it is crucial to re-emphasize the need for more RCTs evaluating the impact of these medications on the incidence and severity of bronchospasm.

Additionally, the manipulation of the larynx and pharynx during the transition from “asleep” to “awake” at tracheal extubation triggers exaggerated neural responses, leading to hemodynamic instability (hypertension and tachycardia) in 10%‒50% of cases.^60^^,^^61^ In this meta-analysis, beta-blockers reduced the incidence of tachycardia and hypertension by 80% and 72%, respectively compared with placebo. This effect may be expected due to the inhibitory action of beta-blockers on adrenergic receptors, which counteracts the effects of sympathetic activation during acute stress, mitigating cardiovascular alterations and nocive events in tracheal extubation.^11^^,^^12^

Patients undergoing tracheal extubation experience a 10%‒30% increase in blood pressure and MHR lasting approximately 5‒15 minutes, which can precipitate various cardiovascular events such as myocardial infarction, arrhythmias, cerebral edema, hemorrhage, and other complications.^17^^,^^61^ Therefore, the use of beta-blockers emerges as a potential intervention to stabilize these hemodynamic parameters, given their ability to mitigate exaggerated sympathetic responses.^11^^,^^12^ This meta-analysis revealed a statistically significant reduction in SBP, DBP, MHR, and MAP with the use of beta-blocker compared to placebo, with the most pronounced mean differences observed within the first 5 minutes post-extubation. Recent RCTs have also demonstrated significant reductions in these hemodynamic outcomes in the intervention group compared to placebo, further supporting the findings of this analysis.^12^^,^^62^ Future studies should focus on optimizing beta-blocker dosing protocols to maximize efficacy while minimizing adverse effects, particularly in patients with preexisting cardiovascular conditions.

The mechanism underlying nausea or vomiting potentially involves the blockade of adrenergic receptors, which can disrupt the cascade of events leading to these adverse events. In some cases, the use of short-acting beta-blockers, such as esmolol, has been shown to effectively manage the hemodynamic fluctuations that can occur during extubation, thereby potentially reducing the incidence of nausea or vomiting.^56^ Our results revealed a 40% reduction in the incidence of nausea and vomiting in patients undergoing tracheal extubation who received beta-blocker compared to those given a placebo. These findings are consistent with previous research, which suggests that beta-blockers can positively impact in incidence of nausea or vomiting.^57^

Our study has some important limitations. Despite our findings showing that beta-blockers effectively reduce hemodynamic complications during extubation, previous studies have reported conflicting results. These discrepancies can be attributed to variations in study design, such as differences in drug dosage, timing of administration (pre-anesthesia vs. intraoperative), and the type of beta-blockers used (e.g., cardioselective vs. non-cardioselective). Additionally, the type of surgery and patient characteristics, such as comorbidities, may affect responses to beta-blockers, with more complex surgeries or patients with cardiovascular issues showing different results. The diversity in anesthetic protocols, particularly the use of pre-anesthetic medications, may also influence outcomes, either masking or enhancing the effects of beta-blockers. Furthermore, discrepancies in outcome measurement, particularly the distinction between “cough” and “bucking” could lead to inconsistent findings. Furthermore, it is important to emphasize that post-extubation cough may also be a consequence of airway manipulation during intubation, which, therefore, represents a limitation in establishing a definitive causal relationship between the observed events. Finally, the primary outcomes were under reported with a greater focus on secondary outcomes. Additionally, the use of univariate meta-regression may have limited the assessment of heterogeneity, as it does not account for potential interactions between covariates. Unfortunately, this limitation exceeds the capacity of the present study to resolve or accurately address. To resolve these inconsistencies, future studies should standardize beta-blocker protocols, patient inclusion criteria, and outcome definitions to provide clearer insights into their hemodynamic benefits during extubation.

## Conclusion

This meta-analysis compared beta-blockers with placebo in 1,803 patients who underwent tracheal extubation. Beta-blockers were associated with lower cough intensity, nausea or vomiting, hypertension, and tachycardia compared with placebo, without significant side effects. These results suggest the potential protective use of these drugs during the peri-extubation period. In this context, their use may be considered to prevent cardiorespiratory responses upon emergence from anesthesia.

This meta-analysis supports the use of beta-blockers to mitigate peri-extubation hemodynamic and airway complications. Further research should focus on defining optimal dosing regimens and identifying patient subgroups who would benefit most from this intervention.

## Appendix Supplementary materials

Image, application 1

## Footnotes

Supplementary material associated with this article can be found in the online version at doi:10.1016/j.bjane.2025.844659.
