# Supplementary material for: Respiratory outcomes of adrenergic beta-antagonists in patients undergoing tracheal extubation: a systematic review and meta-analysis of randomized controlled trials
Source: Braz J Anesthesiol. 2025 Jul 5;75(6):844659. doi: 10.1016/j.bjane.2025.844659 (PMC12345278; doi:10.1016/j.bjane.2025.844659)
Supplement: Supplementary file 1 [file mmc1.docx]

**BJAN-D-24-00651**

**Supplementary material**

**Supplementary Table 1 -** Search strategy.

**Supplementary Table 2 -** Summary of their key characteristics and relevance in the context of tracheal extubation based on the studies included in this meta-analysis.

**Supplementary Table 3 -** Table with the results of comparison between beta-blockers and placebo for SBP, DBP, MAP, and MHR.

**Supplementary Table 4 -** Table with the results of the meta-regression for outcomes of SBP, DBP, MAP, and MHR.

**Supplementary Table 5 -** Traffic light plot with Risk of bias summary for randomized studies (Rob-2).

**Supplementary Figure 1 -** Forest plot comparing beta-blockers with placebo for hypotension.

**Supplementary Figure 2 -** Forest plot comparing beta-blockers with placebo for bradycardia.

**Supplementary Figure 3 -** Forest plot comparing beta-blockers with placebo for hypertension.

**Supplementary Figure 4 -** Forest plot comparing beta-blockers with placebo for tachycardia.

**Supplementary Figure 5 -** Forest plot comparing beta-blockers with placebo for nausea or vomiting.

**Supplementary Figure 6 -** Leave-one-out analyses of the outcome of bucking.

**Supplementary Figure 7 -** Leave-one-out analyses of the outcome of hypertension.

**Supplementary Figure 8 -** Leave-one-out analyses of the outcome of cough.

**Supplementary Figure 9 -** Leave-one-out analyses of the outcome of no/mild cough.

**Supplementary Figure 10 -** Leave-one-out analyses of the outcome of tachycardia.

**Supplementary Table 5 -** Table with Risk of bias summary for randomized studies (RoB 2).

**Supplementary Figure 11** - Funnel Plot with enhanced contour for the outcomes of SBP, DBP, MAP, and MHR and their respective subgroups that had 10 or more studies.

**Supplementary Table 6 -** Egger’s test for outcomes of SBP, DBP, MAP, and MHR.

**Supplementary Table 1 -** Search strategy.

| **Database** | **Search strategy** |
| --- | --- |
| Pubmed | (Esmolol OR Acebutolol OR Atenolol OR Betaxolol OR Bevantolol OR Bisoprolol OR Celiprolol OR Metoprolol OR Practolol OR Landilol OR Butoxamine OR Propranolol OR Carvedilol OR Nebivolol OR Timolol OR Pindolol OR Nadolol OR Sotalol OR Oxprenolol OR Levobunolol OR Penbutolol OR Alprenolol OR Talinolol OR Practolol OR Tertatolol OR Arotinolol OR Bufuralol OR Betaxolol OR Mepindolol OR Xamoterol OR Sotalol OR “beta blocker" OR "beta block" OR "beta blockers" OR "Adrenergic beta Antagonists" OR “betadrenergic Blocker” OR “beta Adrenergic Antagonist” OR “Adrenergic beta Receptor Blockader”) AND (extubation OR extubations) |
| Embase | (Esmolol OR Acebutolol OR Atenolol OR Betaxolol OR Bevantolol OR Bisoprolol OR Celiprolol OR Metoprolol OR Practolol OR Landilol OR Butoxamine OR Propranolol OR Carvedilol OR Nebivolol OR Timolol OR Pindolol OR Nadolol OR Sotalol OR Oxprenolol OR Levobunolol OR Penbutolol OR Alprenolol OR Talinolol OR Practolol OR Tertatolol OR Arotinolol OR Bufuralol OR Betaxolol OR Mepindolol OR Xamoterol OR Sotalol OR “beta blocker" OR "beta block" OR "beta blockers" OR "Adrenergic beta Antagonists" OR “betadrenergic Blocker” OR “beta Adrenergic Antagonist” OR “Adrenergic beta Receptor Blockader”) AND (extubation OR extubations) |
| Cochrane central | (Esmolol OR Acebutolol OR Atenolol OR Betaxolol OR Bevantolol OR Bisoprolol OR Celiprolol OR Metoprolol OR Practolol OR Landilol OR Butoxamine OR Propranolol OR Carvedilol OR Nebivolol OR Timolol OR Pindolol OR Nadolol OR Sotalol OR Oxprenolol OR Levobunolol OR Penbutolol OR Alprenolol OR Talinolol OR Practolol OR Tertatolol OR Arotinolol OR Bufuralol OR Betaxolol OR Mepindolol OR Xamoterol OR Sotalol OR “beta blocker" OR "beta block" OR "beta blockers" OR "Adrenergic beta Antagonists" OR “betadrenergic Blocker” OR “beta Adrenergic Antagonist” OR “Adrenergic beta Receptor Blockader”) AND (extubation OR extubations) |

**Supplementary Table 2 -** Summary of Beta-Blockers Used in the Included Studies and Their Relevance in Tracheal Extubation.

| **Beta-blocker** | **Selectivity** | **Half-life** | **Onset** | **Route** | **Main Findings in Included RCTs** | **Clinical Remarks** |
| --- | --- | --- | --- | --- | --- | --- |
| Esmolol | β1-selective | ~9 min | Rapid | IV | ↓ Cough,  ↓ Hypertension,  ↓ HR (12,13,18) | Ultrashort-acting, ideal for intraoperative control |
| Metoprolol | β1-selective | 3–7 h | Intermediate | IV/Oral | ↓ Bucking, ↓ HR (34) | Longer-acting, effective in preventing reflex responses |
| Landiolol | β1-selective | ~4 min | Rapid | IV | ↓ HR and MAP (23,30) | Esmolol alternative with favorable tolerability |
| Labetalol | Non-selective (β1/β2 + α1) | 5–8 h | Intermediate | IV | ↓ Hypertension (11,35,36) | May help with BP control; use with caution in asthmatics |
| Propranolol | Non-selective | 3–6 h | Intermediate | Oral/IV | ↓ Stress response (29) | Less used in this context; bronchospasm risk possible |

↓ = reduction; HR = heart rate; MAP = mean arterial pressure; BP = blood pressure; RCT = randomized controlled trial; IV = intravenous; min = minutes; h = hours; β1 = beta-1 adrenergic receptor; β2 = beta-2 adrenergic receptor; α1 = alpha-1 adrenergic receptor.

**Supplementary Table 3 -** Table with the results of SBP, DBP, MAP, and MHR. K = number of studies; n.e = number of intervention group patients; n.c = number of control group patients; MD = mean difference; MD.lb = lower bound of the mean difference 95% Confidence Interval (CI); MD.ub = upper bound of the mean difference 95% CI; I^2^ = I^2^ statistics for heterogeneity.

| Outcome | Time | K | n.e | n.c | MD | MD.lb | MD.ub | p-value | I^2^ |
| --- | --- | --- | --- | --- | --- | --- | --- | --- | --- |
| SBP | At Extubation | 6 | 174 | 180 | -16.25 | -27.87 | -4.63 | 0.006 | 0.99 |
|  | 1 minute | 9 | 260 | 216 | -18.99 | -23.94 | -14.04 | <0.001 | 0.75 |
|  | 2 minutes | 2 | 70 | 50 | -18.04 | -24.24 | -11.83 | <0.001 | 0.33 |
|  | 5 minutes | 10 | 270 | 226 | -13.61 | -18.74 | -8.48 | <0.001 | 0.86 |
|  | 10 minutes | 8 | 246 | 208 | -5.91 | -10.17 | -1.64 | 0.007 | 0.79 |
|  | 15 minutes or more | 4 | 116 | 122 | -5.91 | -9.3 | -2.52 | 0.001 | 0.72 |
| DBP | At Extubation | 4 | 98 | 98 | -13.95 | -24.71 | -3.18 | 0.011 | 0.97 |
|  | 1 minute | 8 | 215 | 171 | -12.62 | -18.7 | -6.54 | <0.001 | 0.91 |
|  | 2 minutes | 2 | 70 | 50 | -12.07 | -14.24 | -9.89 | <0.001 | 0 |
|  | 5 minutes | 9 | 225 | 181 | -7.5 | -11.08 | -3.92 | <0.001 | 0.76 |
|  | 10 minutes | 6 | 170 | 126 | -4.45 | -6.04 | -2.86 | <0.001 | 0.03 |
|  | 15 minutes or more | 3 | 85 | 85 | -4.62 | -7.18 | -2.07 | <0.001 | 0.38 |
| MHR | At Extubation | 10 | 298 | 303 | -19.81 | -28.08 | -11.55 | <0.001 | 0.98 |
|  | 1 minute | 11 | 315 | 271 | -22.51 | -32.21 | -12.82 | <0.001 | 0.98 |
|  | 2 minutes | 2 | 70 | 50 | -30.29 | -51.54 | -9.05 | 0.005 | 0.98 |
|  | 5 minutes | 14 | 448 | 339 | -15.47 | -20.3 | -10.63 | <0.001 | 0.96 |
|  | 10 minutes | 9 | 261 | 208 | -9.95 | -10.89 | -9.01 | <0.001 | 0.41 |
|  | 15 minutes or more | 5 | 131 | 137 | -5.18 | -11.26 | 0.89 | 0.094 | 0.96 |
| MAP | At Extubation | 8 | 304 | 306 | -9.94 | -17.15 | -2.72 | 0.007 | 0.99 |
|  | 1 minute | 6 | 244 | 227 | -12.64 | -17.52 | -7.76 | <0.001 | 0.98 |
|  | 2 minutes | 2 | 70 | 50 | -13.64 | -15.41 | -11.88 | <0.001 | 0 |
|  | 5 minutes | 12 | 471 | 365 | -6.99 | -10.65 | -3.32 | <0.001 | 0.87 |
|  | 10 minutes | 7 | 272 | 216 | -2.2 | -2.81 | -1.59 | <0.001 | 0.63 |
|  | 15 minutes or more | 5 | 110 | 109 | -3.85 | -9.25 | 1.55 | 0.162 | 0.81 |

**Supplementary Table 4 -** Table with the results of the meta-regression for outcomes of SBP, DBP, MAP, and MHR using the mean age and the mean values at baseline of SBP, DBP, MAP, and MHR as predictors. K = number of studies; b = beta of the regression; LB = lower bound of the beta’s 95% Confidence Interval (CI); UB = upper bound of the beta’s 95% CI; QMp = p-value for the test of moderators; I^2^ = I^2^ statistics for heterogeneity; R2 = explained variance of the predictor.

| Outcome | Time | Predictor | K | b | LB | UB | QMp | I^2^ | R2 |
| --- | --- | --- | --- | --- | --- | --- | --- | --- | --- |
| SBP | 1 minute | AGE | 9 | -0.03 | -0.54 | 0.48 | 0.907 | 76.99 | 0 |
|  |  | SBP | 9 | 0.05 | -0.4 | 0.5 | 0.821 | 76.74 | 0 |
|  |  | DBP | 8 | -0.9 | -2.59 | 0.79 | 0.298 | 78.33 | 0 |
|  |  | MHR | 10 | 0.24 | -0.97 | 1.44 | 0.699 | 96.86 | 0 |
|  | 5 minutes | AGE | 9 | -0.33 | -0.93 | 0.26 | 0.27 | 80.16 | 0 |
|  |  | SBP | 10 | -0.42 | -0.96 | 0.13 | 0.132 | 83.5 | 15.01 |
|  |  | DBP | 9 | -0.33 | -2.14 | 1.47 | 0.716 | 88.97 | 0 |
|  |  | MHR | 9 | 0.31 | -0.45 | 1.06 | 0.426 | 87.22 | 0 |
| DBP | 5 minutes | SBP | 9 | -0.17 | -0.63 | 0.28 | 0.451 | 79.66 | 0 |
|  |  | DBP | 9 | -0.45 | -1.55 | 0.64 | 0.417 | 79.77 | 0 |
| MAP | 5 minutes | AGE | 12 | -0.28 | -0.69 | 0.13 | 0.183 | 93.06 | 6.7 |
|  |  | MAP | 12 | 0.4 | -0.02 | 0.83 | 0.061 | 91.57 | 27.89 |
|  |  | MHR | 11 | 0.03 | -0.32 | 0.38 | 0.870 | 93.66 | 0 |
| MHR | At Extubation | AGE | 10 | 0.91 | 0.41 | 1.4 | <0.001 | 95.54 | 58.76 |
|  |  | MHR | 10 | 0.36 | -0.69 | 1.41 | 0.501 | 97.69 | 0 |
|  | 1 minute | AGE | 11 | 0.42 | -0.56 | 1.4 | 0.4 | 0 | 97.43 |
|  |  | SBP | 10 | 0.24 | -0.71 | 1.19 | 0.626 | 0 | 97.36 |
|  |  | MHR | 11 | 0.32 | -0.85 | 1.49 | 0.592 | 0 | 97.49 |
|  | 5 minutes | AGE | 14 | 0.09 | -0.26 | 0.44 | 0.603 | 93.35 | 0 |
|  |  | SBP | 9 | -0.04 | -0.73 | 0.65 | 0.905 | 92.51 | 0 |
|  |  | MAP | 10 | 0.13 | -0.59 | 0.86 | 0.716 | 93.19 | 0 |
|  |  | MHR | 14 | -0.33 | -0.77 | 0.1 | 0.136 | 92.06 | 10.16 |

**Supplementary Figure 1 -** There was no difference between beta-blockers and placebo in the incidence of hypotension in patients undergoing orotracheal extubation. MH = Mantel-Haenszel; CI = Confidence Interval.


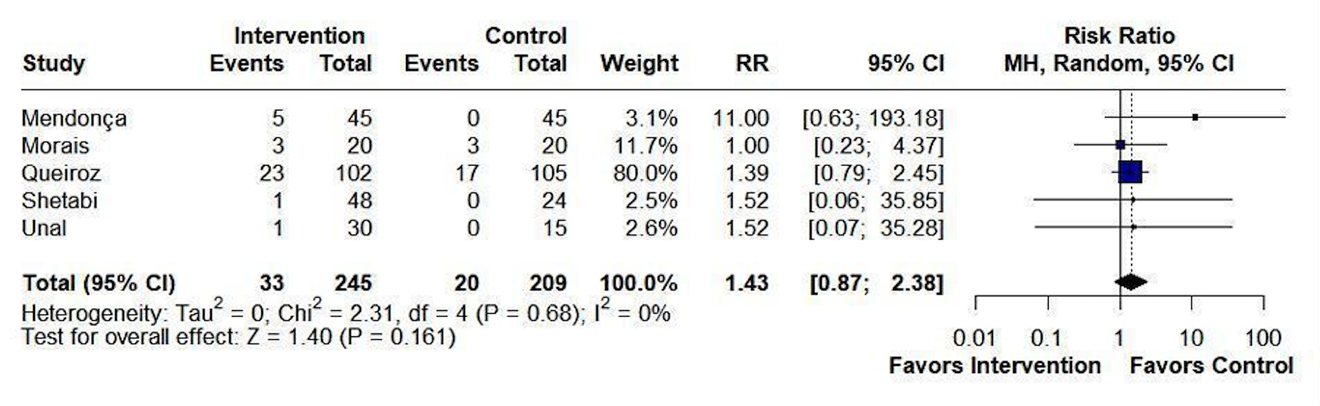


**Supplementary Figure 2 -** There was no difference between beta-blockers and placebo in the incidence of bradycardia in patients undergoing orotracheal extubation. MH = Mantel-Haenszel; CI = Confidence Interval.


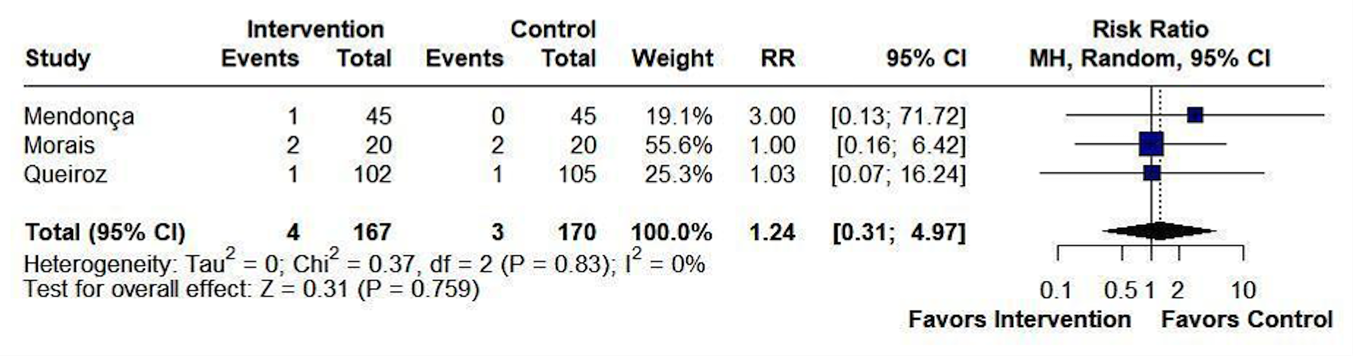


**Supplementary Figure 3 -** Beta-blockers significantly reduced the incidence of hypertension in patients undergoing orotracheal extubation. MH = Mantel-Haenszel; CI = Confidence Interval.


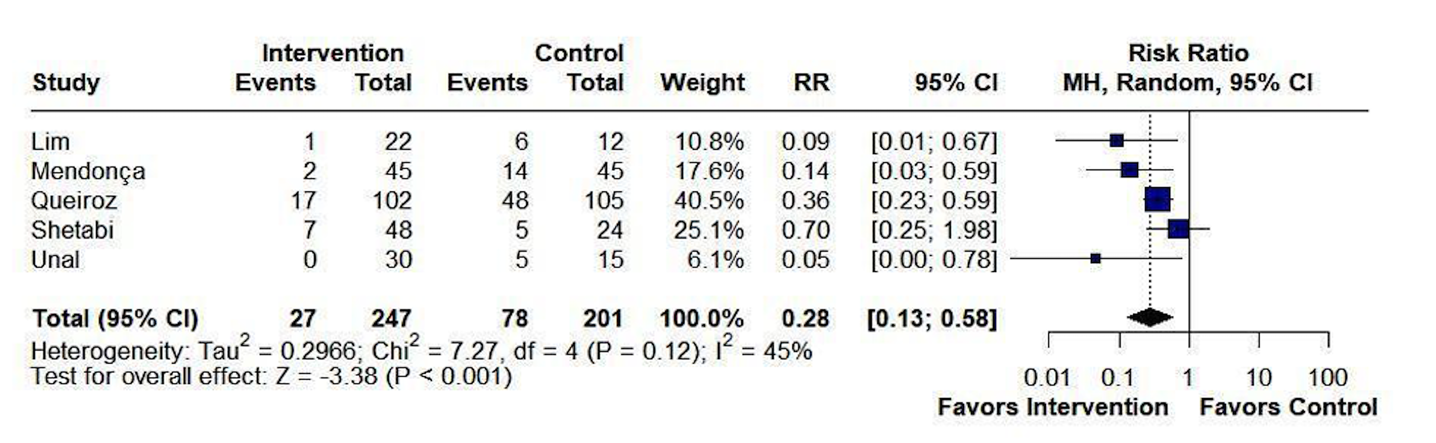


**Supplementary Figure 4 -** Beta-blockers significantly reduced the incidence of tachycardia in patients undergoing orotracheal extubation. MH = Mantel-Haenszel; CI = Confidence Interval.


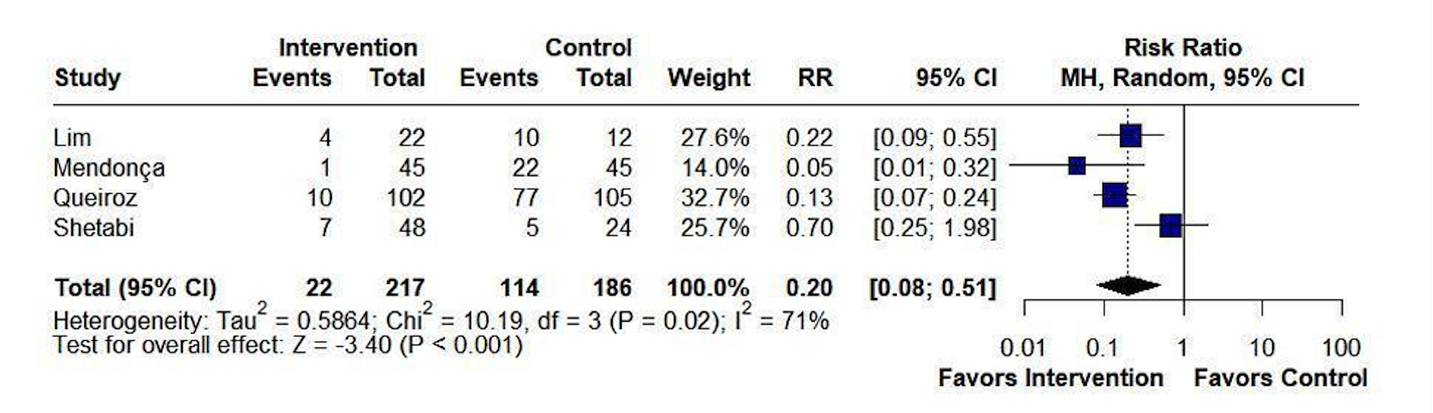


**Supplementary Figure 5 -** Beta-blockers significantly reduced the incidence of nausea or vomiting in patients undergoing orotracheal extubation. MH = Mantel-Haenszel; CI = Confidence Interval.


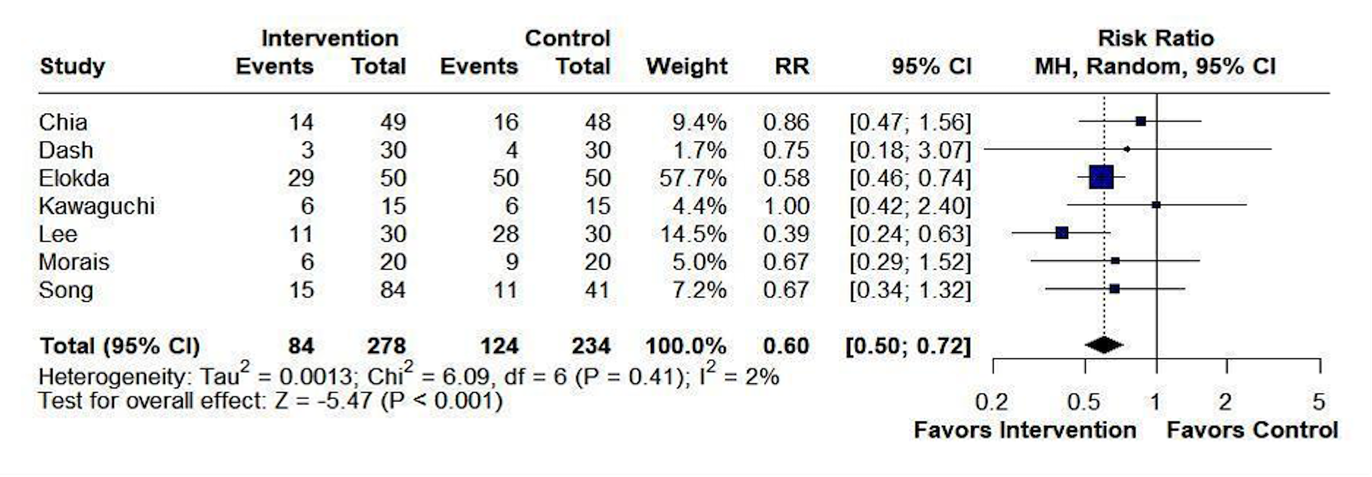


**Supplementary Figure 6 -** Leave-one-out analyses of the outcome of bucking showed that the omission of the study Mendonça (2023) rendered the highest reduction in heterogeneity.


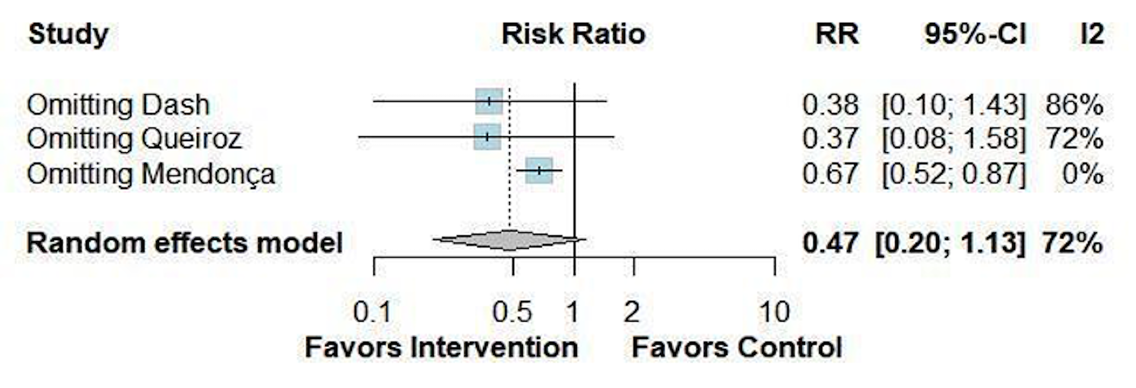


**Supplementary Figure 7 -** Leave-one-out analyses of the outcome of hypertension showed that the omission of the study Shetabi (2023) rendered the lowest heterogeneity possible.


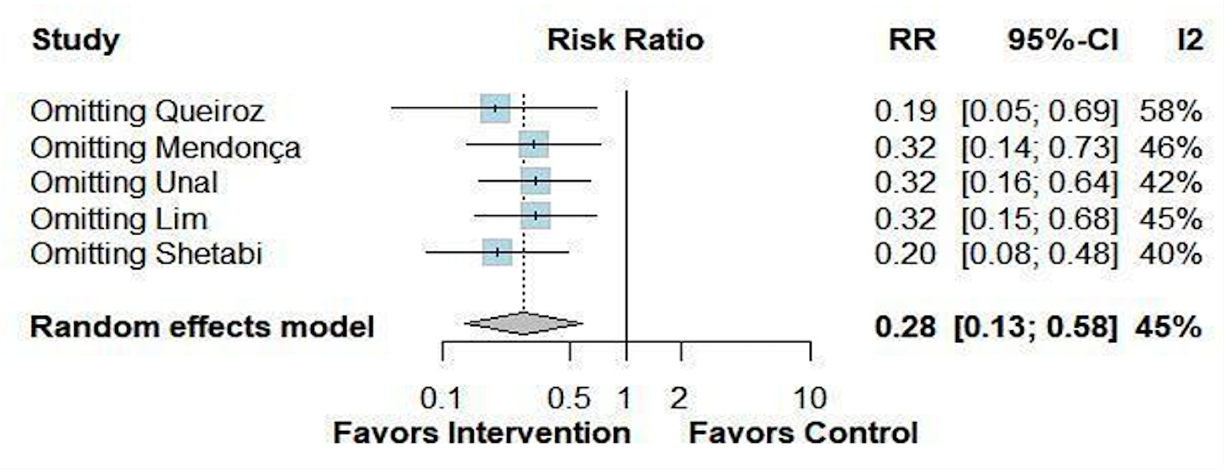


**Supplementary Figure 8 -** Leave-one-out analyses of the outcome of cough showed that the omission of the study Mendonça (2023) rendered the lowest heterogeneity possible.


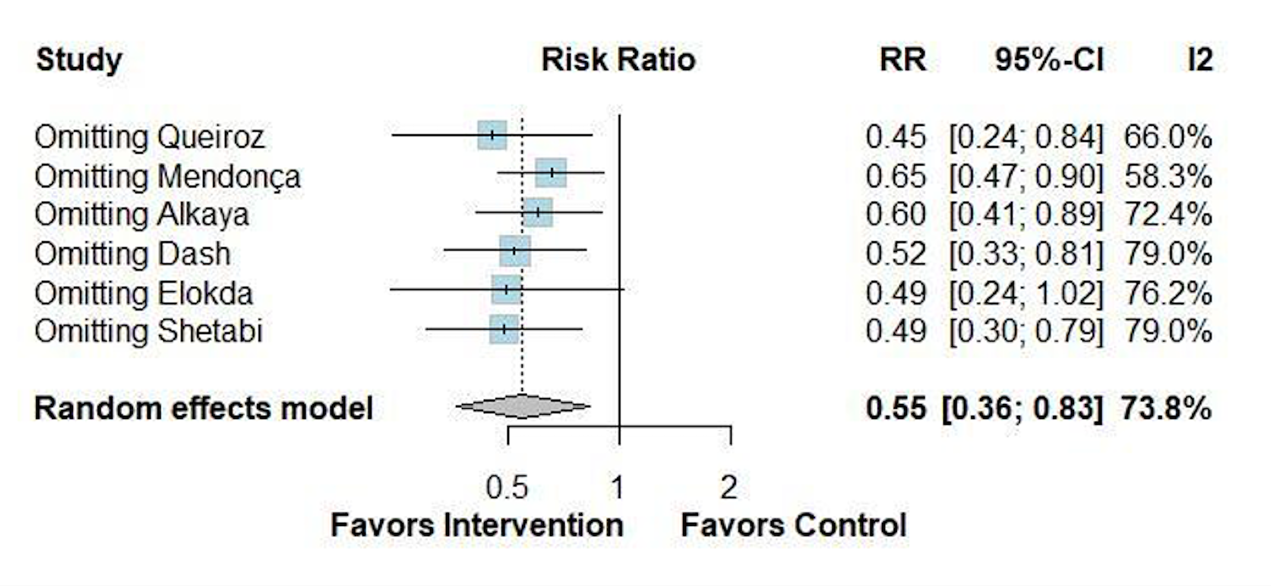


**Supplementary Figure 9 -** Leave-one-out analyses of the outcome of no or mild cough showed that the omission of the study Elokda (2015) rendered the lowest heterogeneity possible.


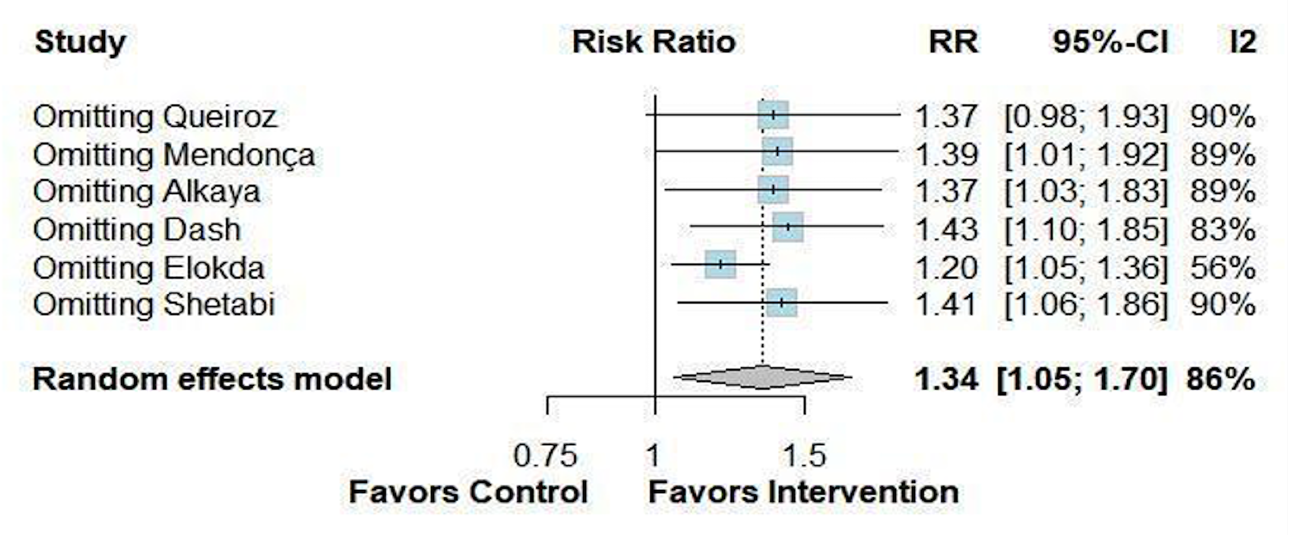


**Supplementary Figure 10 -** Leave-one-out analyses of the outcome of tachycardia showed that the omission of the study Shetabi (2023) rendered the lowest heterogeneity possible.


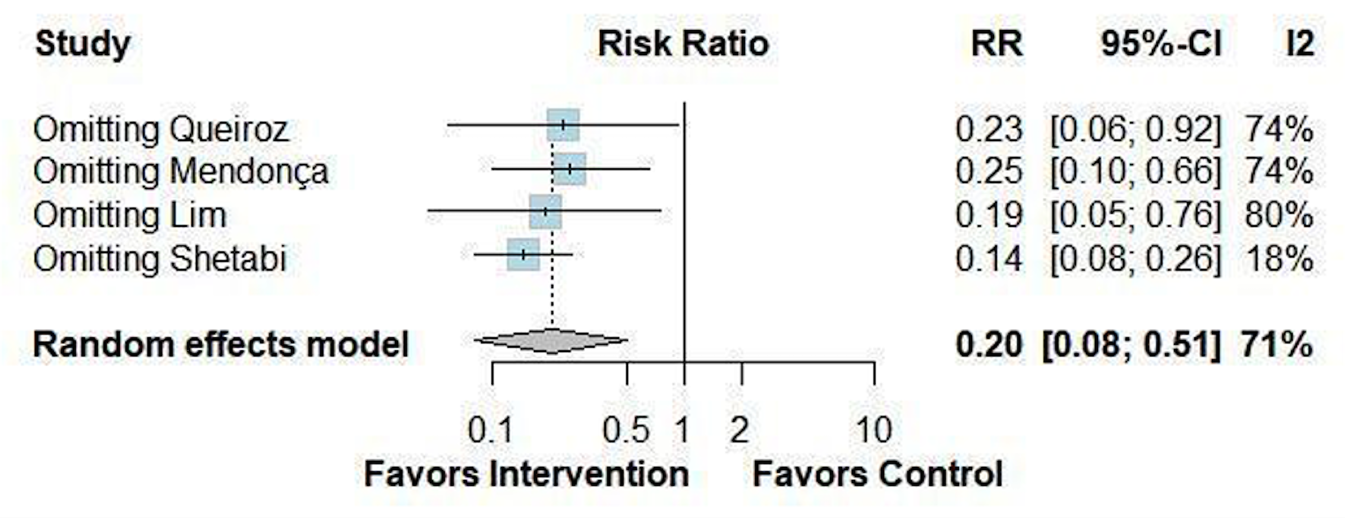


**Supplementary Table 5 -** Traffic light plot with Risk of bias summary for randomized studies (Rob-2).

**
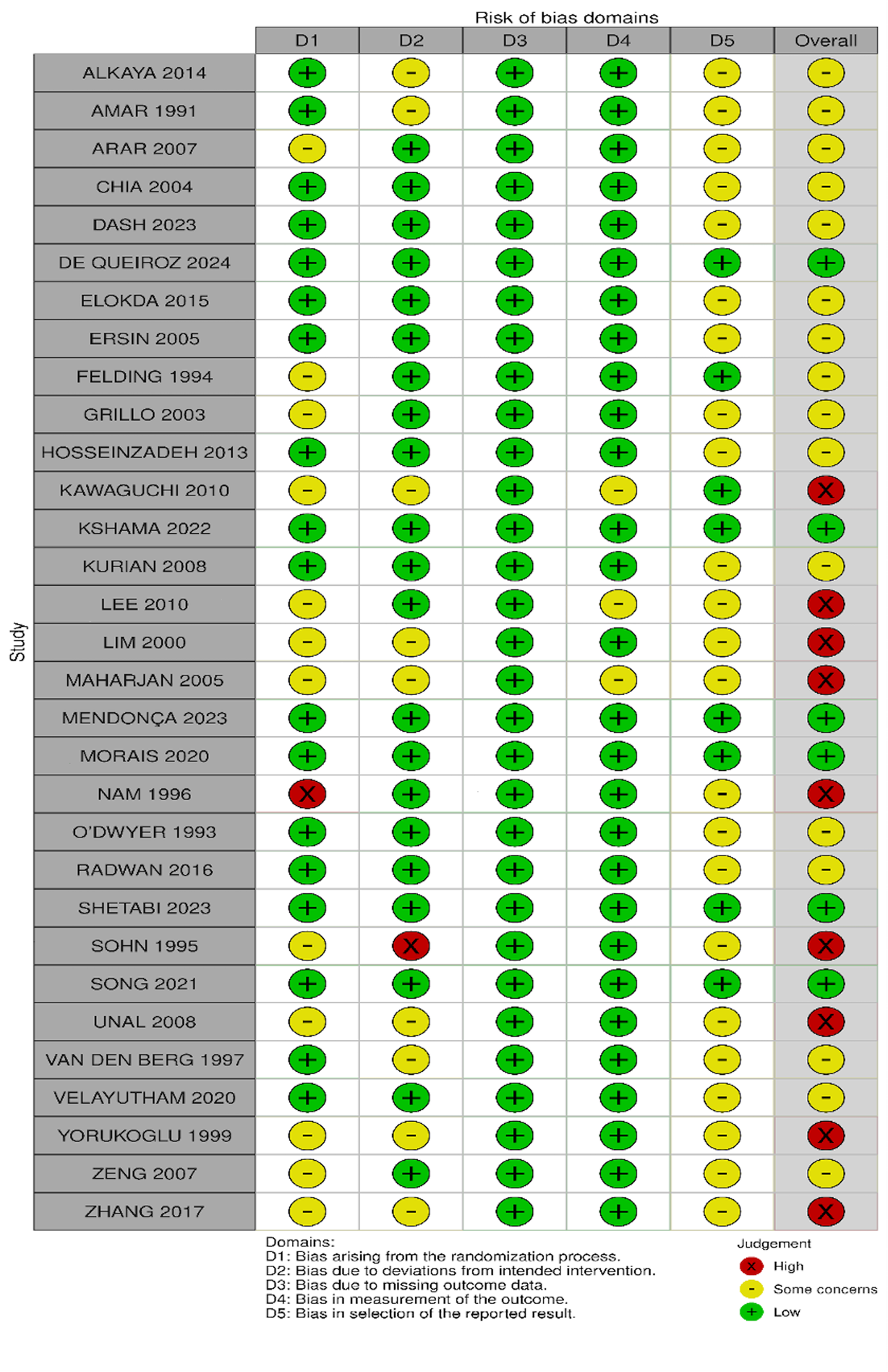
**

**Supplementary Figure 11 -** Funnel Plot with enhanced contour for the outcomes of SBP, DBP, MAP, and MHR and their respective subgroups that had 10 or more studies. Mean Difference in the X axis versus Standard Error in the Y axis. The contour on the plots indicates studies that fall under a certain threshold of p-values (< 0.1; < 0.05; < 0.01).

**
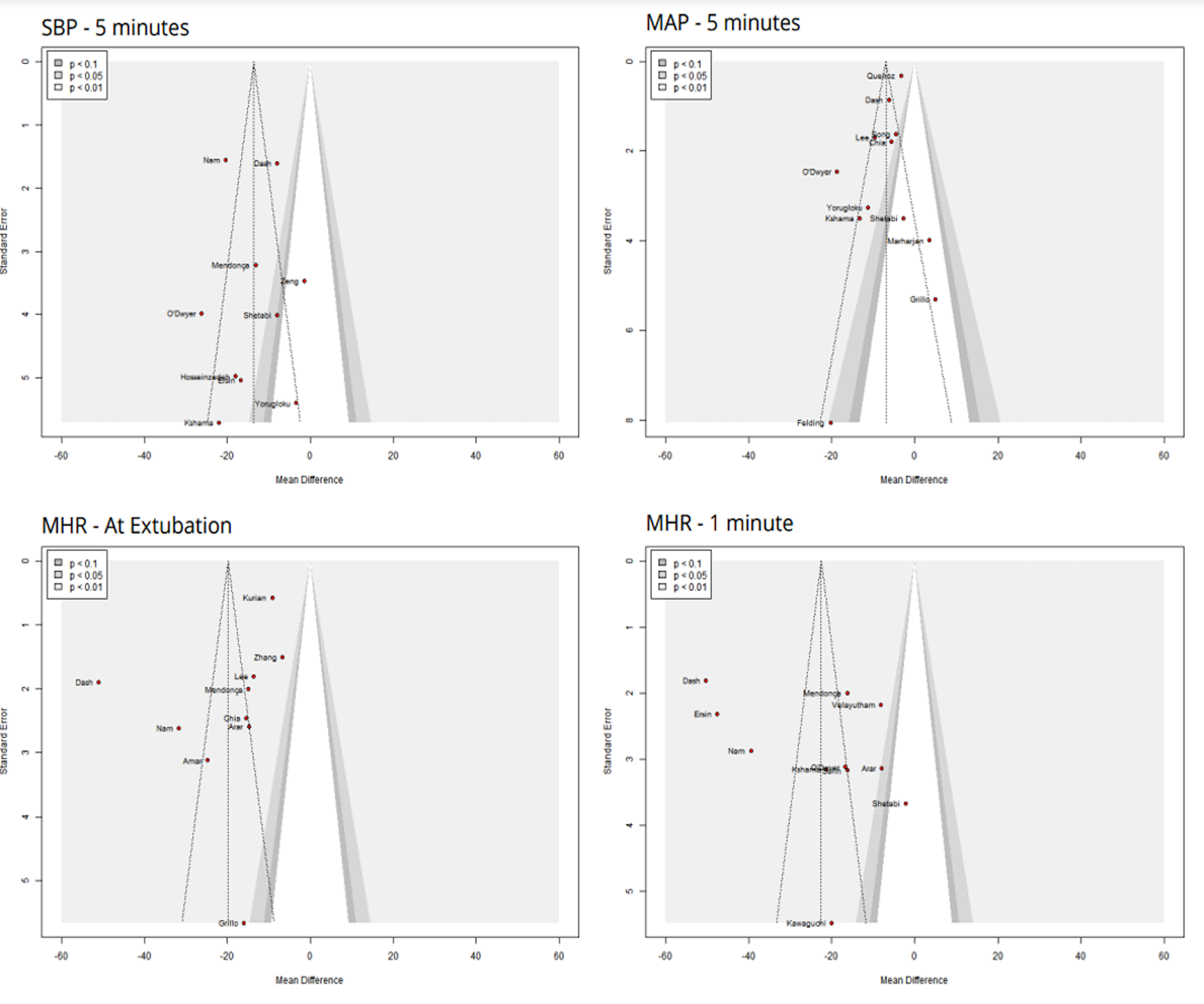
**

**
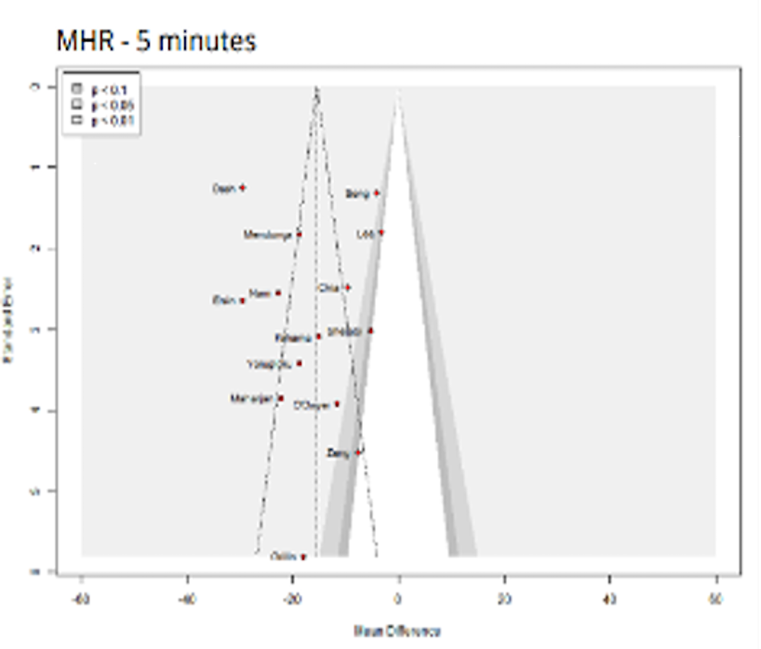
**

**Supplementary Table 6 -** Table with the results of the egger’s test for outcomes of SBP, DBP, MAP, and MHR. LB = lower bound of the intercept’s 95% Confidence Interval (CI); UB = upper bound of the intercept’s 95% CI; t = t statistics of the Egger’s test; p = p-value for the Egger’s test.

| Outcome | Time | Intercept | LB | UB | t | p |
| --- | --- | --- | --- | --- | --- | --- |
| SBP | 5 minutes | 0.221 | -3.489 | 3.932 | 0.117 | 0.91 |
| MAP | 5 minutes | -1.771 | -3.559 | 0.017 | -1.941 | 0.081 |
| MHR | At Extubation | -6.29 | -13.658 | 1.078 | -1.673 | 0.133 |
|  | 1 minute | 10.137 | -4.159 | 24.432 | 1.39 | 0.198 |
|  | 5 minutes | 0.539 | -5.884 | 6.962 | 0.165 | 0.872 |
